# Identification and Characterization of Canine Parvoviruses and Emergence of Canine Bocavirus and Bufavirus from Diarrheic Dogs in Sichuan Province, China

**DOI:** 10.3390/vetsci13010041

**Published:** 2026-01-02

**Authors:** Siyu Liu, Xiaoqi Li, Yuxin Zhou, Shuangshuang Song, Yuyan Huang, Mengjie Che, Xin Lei, Iram Laghari, Mingyue Wu, Ruilin Han, Haifeng Liu, Ziyao Zhou, Guangneng Peng, Kun Zhang, Zhijun Zhong

**Affiliations:** Key Laboratory of Animal Disease and Human Health of Sichuan, College of Veterinary Medicine, Sichuan Agricultural University, Chengdu 611130, China; llsy6412024@163.com (S.L.);

**Keywords:** canine parvovirus, canine bocavirus, canine bufavirus, VP2, phylogenetic analysis

## Abstract

Canine parvovirus (CPV) is the primary cause of viral enteritis in dogs, while canine bocavirus (CBoV) and canine bufavirus (CBuV) have emerged as significant components of the canine enteric virome. Previous studies on CPV in Sichuan had limited geographic coverage and sample size, and lacked data on CBoV and CBuV. This study used PCR to investigate the genetic diversity of CPV, CBoV and CBuV and to examine their co-infection status in diarrheic dogs across five regions of Sichuan between 2020 and 2022. The results revealed that CPV-2 was the most prevalent virus (33.3%, 48/144), while CBoV (5.56%, 8/144) and CBuV (4.17%, 6/144) were detected for the first time in Sichuan province. Genetic analysis revealed CPV-2c to be the predominant genotype (95.8% (46/48)), replacing the previously circulating strains CPV-2a and CPV-new 2a. The study also identified several typical and novel mutations in the VP2 protein in the CPV-2c strains, including Ala5Gly, Trp214Cys and Thr440Ala. Additionally, four co-infection cases (2.78%) were observed, including a triple CPV/CBoV/CBuV infection in Xichang (XC05). Phylogenetic analysis revealed genetic diversity, with CBoV strains clustering into CBoV-1 and CBoV-2 subspecies, while CBuV strains formed two distinct clusters. Our findings emphasize the need for ongoing monitoring of the dynamic epidemiological situation of CPV, CBoV and CBuV in Sichuan Province.

## Simple Summary

Canine diarrhea is frequently associated with viral infections. While canine parvovirus (CPV) is a well-known pathogen, two recently discovered viruses—canine bocavirus (CBoV) and canine bufavirus (CBuV)—are also recognized as potential contributors. This study investigated the presence and characteristics of these three viruses in diarrheic dogs from five regions in Sichuan, China. Our findings showed that CPV-2 (33.3%, 48/144) was the most prevalent virus. Notably, this is the first report of CBoV (5.56%, 8/144) and CBuV (4.17%, 6/144) detection in Sichuan. The dominant CPV strain identified was CPV-2c, which had replaced previously circulating strains such as CPV-2a and new CPV-2a. We also identified novel genetic mutations and documented several co-infection cases where dogs were infected with multiple viruses. These results reveal a dynamic and evolving viral situation in Sichuan’s dog population, highlighting the importance of ongoing surveillance to effectively manage disease spread and protect canine health.

## 1. Introduction

Canine infectious diarrhea poses a considerable challenge to veterinary practitioners due to the multifaceted etiology of the condition, which encompasses viral infections, parasitic infections and gut microbiota dysbiosis [1,2,3]. Among the viral pathogens, canine parvovirus (CPV), canine bocavirus (CBoV) and canine bufavirus (CBuV), all belonging to the genus Protoparvovirus, are three major enteric viruses of concern. CPV-2 has been identified as the primary causative agent of severe viral diarrhea, particularly in puppies [4]. CBoV and CBuV are newly discovered parvoviruses that have also been associated with canine diarrhea and require further investigation into their epidemiology [5,6]. Co-infections involving the three viruses are commonly observed and may exacerbate disease progression, leading to more severe clinical manifestations [7,8].

CPV is a single-stranded DNA virus that contains two large open reading frames (ORFs). It has been established that ORF1 encodes two non-structural proteins, designated as NS1 and NS2, while ORF2 encodes two structural proteins, termed VP1 and VP2 [9,10]. VP2, the major capsid protein of CPV, is the primary determining factor in genotype classification, based on variations at key antigenic sites, notably residues 426 and 297 [11]. The continuous molecular evolution of the key VP2 residues has driven the emergence and dominance of new antigenic variants in China. Three antigenic variants—CPV-2a (426Asn), CPV-2b (426Asp), and CPV-2c (426Glu)—are determined by variations at residue 426 of the VP2, while novel strains (e.g., new CPV-2a/2b) are distinguished by Ser297Ala [12,13,14]. To date, all major CPV-2 genotypes (including CPV-2a, -2b, -2c, and new variants) have been identified across China, with the dominant CPV genotypes exhibiting variation across Chinese provinces. It has been reported that CPV-2c is the predominant strain in Shanghai, Shandong, Tangshan, Jilin, Guangxi, and Henan provinces [15,16,17,18,19,20,21]. Meanwhile, CPV-2a and new CPV-2a have shown prominence in regions including Gansu, as well as in Heilongjiang and Jiangsu, respectively [22,23,24]. In Sichuan, only four studies have focused on the molecular epidemiology of CPV, and these studies are constrained by several limitations, including small sample sizes (e.g., samples number less than 58) and a restricted geographical focus (often limited to Chengdu, the capital of Sichuan Province) [25,26,27,28].

CBoV and CBuV are recently discovered parvoviruses in China that are associated with gastroenteritis of varying severity in canines [29,30]. Of the three genotypes of CBoV (CBoV-1, -2, and -3) that have been reported on a global scale, CBoV-2 has been confirmed as enteropathogenic [31,32,33,34,35]. In China, CBoV has been reported in Heilongjiang [36], Guangzhou [31] and Hong Kong [37]. The first documented detection of CBuV was in dogs with intestinal and respiratory tract disease in Italy in 2018 [38]. Subsequent reports have emerged from China [39,40], India [41] and Turkey [42]. In China, CBuV was first documented in Guangxi in 2019 [39], with subsequent reports from Shanghai, Henan and Anhui [40,43,44]. It is important to note that co-infection with CBuV and CPV have been observed in several studies [39,45]. Furthermore, these viruses have also been identified from apparently healthy animals [46]. However, further research is required to elucidate the molecular epidemiology of CBoV and CBuV in Sichuan Province.

According to the 2025 China Pet Industry White Paper, the number of pet dogs in China was 52.58 million in 2024 [47]. Sichuan, a province with a substantial population, is characterized by a significant canine population. At present, molecular epidemiological data and co-infection patterns on CBoV, CBuV and CPV remain limited and require updating in Sichuan Province. To address these knowledge gaps, the aim of the present study was to investigate the characteristics of CPV, CBoV and CBuV in Sichuan province, and also investigated their co-infection status.

## 2. Materials and Methods

A total of 144 fecal samples were collected from 144 dogs that presented with diarrhea and accompanying clinical signs (including fever, vomiting, anorexia, or lethargy) to local veterinary clinics for clinical care. All the samples were collected from five districts in Sichuan Province between November 2020 and June 2022. The clinical symptoms and vaccination history of each dog were recorded at the time of sampling. Samples were collected with an anal swab and placed into a 1.5 mL centrifuge tube containing 1.0 mL of phosphate-buffered saline (PBS). The samples were obtained through convenience sampling of clinically affected dogs presented for veterinary care. The samples were sourced from five distinct regions with a high density of domestic dogs: Chengdu (n = 44), Ya’an (n = 35), Mianyang (n = 21), Leshan (n = 29), and Xichang (n = 15). All samples were stored at −20 °C until further processing.

Total viral DNA was extracted from fecal samples using the QIAamp PowerFecal Pro DNA Kit (Qiagen Co., Ltd., Hilden, Germany) in accordance with the manufacturer’s protocols. The extracted DNA was then amplified using polymerase chain reaction (PCR) with previously published primer sets targeting specific genes: the complete VP2 gene of CPV-2 (1755 bp), the NS1 gene of CBoV (440 bp), and the VP2 gene of CBuV (962 bp) [23,25,48]. The primer sequences used are detailed in Table S1. The reactions were performed in a total volume of 50 µL (for CPV and CBoV) or 25 µL (for CBuV) under three different reaction conditions (Table S1). The amplification products of the genes were sent to Sangon Biotech (Shanghai) Co., Ltd., Shanghai, China for bidirectional sequencing analysis.

The obtained gene sequences (the complete VP2 gene of CPV, the NS1 gene of CBoV and the VP2 gene of CBuV) were initially analyzed using the BLAST web interface (National Center for Biotechnology Information, Bethesda, MD, USA; https://blast.ncbi.nlm.nih.gov/Blast.cgi, accessed on 15 May 2024) to identify and download the reference sequences (95 strains of CPV, 16 strains of CBoV, and 23 strains of CBuV) from the GenBank database. Multiple sequence alignments for each virus were performed using MEGA software (version 11.0; Mega Limited, Auckland, New Zealand). Specifically, the phylogenetic tree for CPV was reconstructed using the Maximum Likelihood method based on the Tamura 3-parameter model with gamma distribution, and the trees for CBoV and CBuV were constructed using the Neighbor-Joining method with the Kimura 2-parameter model. The robustness of the nodes was tested using 1000 bootstrap replications and bootstrap support values greater than 60 were shown at the nodes. Additionally, the VP2 genes of CPV and CBuV, along with the NS1 gene of CBoV, were translated into amino acid sequences using MEGA (version 11.0). The nucleotide and amino acid sequence identities between the isolates from this study and the reference strains included in the phylogenetic trees were calculated using the MegAlign module within the BioEdit software (version 7.2.5; Ibis Biosciences, Carlsbad, CA, USA).

The calculation of detection rates and the subsequent determination of 95% confidence intervals (95% CI) was conducted utilizing the statistical software SPSS 22.0 for Windows (SPSS Inc., Chicago, IL, USA).

## 3. Results

### 3.1. Distribution of CPV, CBoV, and CBuV in Diarrheic Dogs

**Figure 1:**
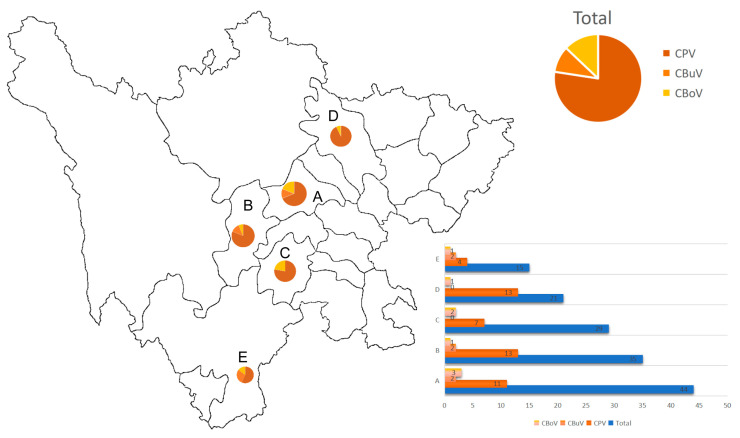
Geographical locations and sample statistics of the isolates analyzed in this study with positive rates of three viruses. In the histogram, the various color bars show the number of sequenced samples. The pie chart illustrates the proportion of each virus detected in the total number of samples analyzed. Note. 1: A: Chengdu city; B: Ya’an city; C: Leshan city; D: Mianyang city; E: Xichang city.

**Figure 2:**
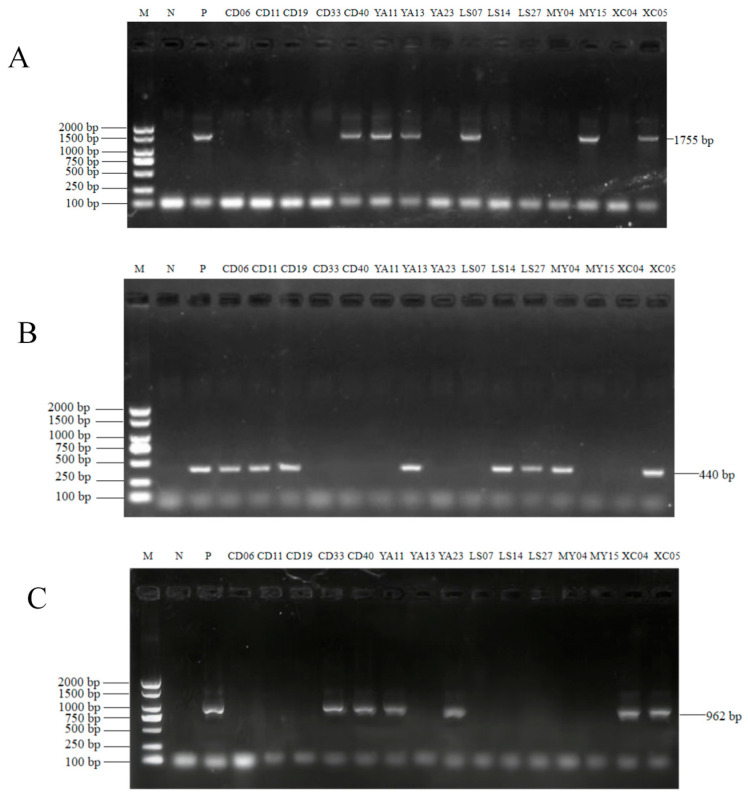
(**A**) Specific fragment of the CPV-2 VP2 gene (1755 bp) was amplified with CPV-VP2’s primer. (**B**) Specific fragment of the CBoV NS1 gene (440 bp) was amplified with CBoV-NS1’s primer. (**C**) Specific fragment of the CBuV VP2 gene (962 bp) was amplified with CBuV-VP2’s primer. Lane M, TaKaRa DL 2000 DNA Marker; lane N, negative control; lane P, positive control.

Among the 144 fecal samples collected from diarrheic dogs across five cities in Si-chuan Province between 2020 and 2022, CPV-2 was the most frequently detected virus (33.3%, 48/144; 95% CI: 25.6–41.0%), followed by CBoV (5.56%, 8/144; 95% CI: 1.8–9.3%) and CBuV (4.17%, 6/144; 95% CI: 0.9–7.4%) (Figure 1, Figure 2 and Figure S1). CPV-2 and CBoV were detected in all five regions, whereas CBuV was only detected in Chengdu (4.55%, 2/44; 95% CI: 0.0–10.7%), Ya’an (5.71%, 2/35; 95% CI: 0.0–13.4%), and Xichang (13.33%, 2/15; 95% CI: 0.0–30.5%). The highest detection rate of CPV-2 was observed in Mianyang (61.9%, 13/21; 95% CI: 41.1–82.7%), whereas the lowest was detected in Leshan (24.13%, 7/29; 95% CI: 8.6–39.7%). For CBoV, the highest detection rate was observed in Leshan (6.7%, 2/29; 95% CI: 0.0–15.8%), while the lowest was detected in Ya’an (2.86%, 1/35; 95% CI: 0.0–8.4%).

Clinical manifestations among virus-positive dogs are summarized in Table 1. Among the 48 CPV-2–positive dogs, 10 dogs exhibited hemorrhagic diarrhea, accounting for 20.8%. In contrast, none of the eight CBoV-positive dogs presented with hemorrhagic diarrhea. Among the six CBuV-positive dogs, only one case (16.7%) was observed with hemorrhagic diarrhea. The vaccination status of virus-positive dogs was also analyzed. Among the 48 CPV-2–positive dogs, 44 (91.67%) had either not received vaccination or had not completed the full vaccination course. Similarly, incomplete or absent vaccination was observed in six of the eight CBoV-positive dogs (75.0%) and in all six CBuV-positive dogs (100%). Based on individual-level clinical and virological data, four cases of co-infection were also identified (Table 1): one case of CPV/CBuV co-infection in Chengdu (CD40); one case of CPV/CBuV and one case of CPV/CBoV co-infection in Ya’an (YA11/13); and one triple CPV/CBoV/CBuV co-infection in Xichang (XC05). All co-infected dogs were young (<1 year) and presented with a consistent set of clinical signs, including diarrhea, lethargy, and anorexia.

### 3.2. Phylogenetic and Genetic Analysis of CPV-2

#### 3.2.1. Amino Acid Analysis Suggests the Predominance of CPV-2c in Sichuan

Of the 48 CPV-2 strains analyzed, 46 (95.8%) were identified as CPV-2c (426-Glu), while two (4.2%) were identified as CPV-new 2a (297-Ala, 426-Asn). These results indicated that CPV-2c was the predominant genotype in Sichuan. The key AA sites for virus evolution were summarized in Table S2. All 46 CPV-2c strains in this study shared three characteristic amino acid substitutions (Phe267Tyr, Tyr324Ile and Gln370Arg) in VP2 with the original CPV-2 reference strain (M38245), which has been documented as a characteristic of CPV-2c strains of Asian origin. The majority (95.65%, 44/46) of the CPV-2c strains retained the traditional alanine (Ala) at VP2 position 5. However, two strains (CD21 and MY20) exhibited an Ala5Gly amino acid mutation. In addition to these typical VP2 amino acid mutations (Ala5Gly, Phe267Tyr, Tyr324Ile and Gln370Arg), several novel mutations were identified. These included a Met447Ile substitution in one CPV-2c strain (LS03), a Thr440Ala mutation in one CPV-2c strain (CD04) and in both CPV-new 2a strains (CD03 and YA06), and a Trp214Cys mutation in four CPV-2c strains from Xichang (XC02, XC05, XC06 and XC08).

#### 3.2.2. VP2 Gene Phylogenetic Analysis Revealed Three Main CPV-2c Clades

**Figure 3:**
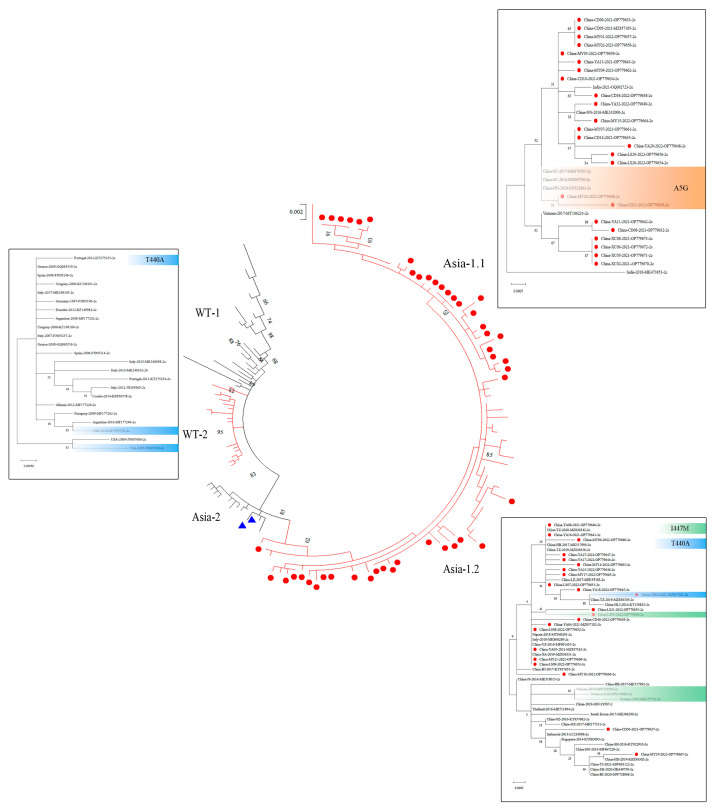
Phylogenetic tree of canine parvovirus (CPV) constructed based on the complete VP2 gene sequences. The tree was inferred using the Maximum Likelihood method with the Tamura 3-parameter model. A discrete Gamma distribution (5 categories, +G, parameter = 0.1000) was used to model evolutionary rate differences among sites. The tree is drawn to scale, with branch lengths measured in the number of substitutions per site. The analysis involved 138 nucleotide sequences, and there was a total of 1755 positions in the final dataset after the removal of all positions containing gaps and missing data (complete deletion option). Red circles indicate CPV-2c strains identified in the present study, and blue triangles indicate CPV-2a strains identified in this study.

The phylogenetic relationship of CPV-2 was reconstructed based on 143 complete VP2 gene sequences, including the 48 strains from this study. The phylogenetic tree showed that all sequences clustered into two major clades defined by key residues at VP2 positions 267 and 324. One clade was characterized by the amino acid substitutions Phe267Tyr/Tyr324Ile, predominantly found in Asian strains, while the other clade exhibited the residues Phe267/Tyr324, predominantly found in Western strains (Figure 3, Table S2). Five primary monophyletic groups were identified: Asia-2, Asia-1.1, Asia-1.2, WT-1 and WT-2. All CPV-2c strains from this study were grouped within the Asia-1.1 and Asia-1.2 subclades. In contrast, the two CPV-new 2a strains (CD03 and YA06) clustered within the Asia-2 subclade.

The Asia-1.1 and Asia-1.2 subclades were differentiated by the residue at VP2 position 447 (Met/Ile). A total of 24 CPV-2c strains in this study were found to cluster within the Asia-1.1. The MY20 and CD21 strains showed a close phylogenetic relationship with three reference strains from China: two from Sichuan (MH476585 and MF805796) and one from Henan (ON322803). Notably, the amino acid sequence of the MY20 strain was 100% identical to that of these three reference strains. The Asia-1.2 subclade comprised CPV-2c strains from China (including 22 strains from this study), Vietnam, Thailand, South Korea, and Indonesia. Within the Asia-1.2 subclade, LS03 strain in our study and three Vietnamese strains (MW239584, MW239603 and MK357721) all carried the 447M residue but were located on different small branches. Another strain from this study (CD04) clustered with Chinese CPV-2c mutants (MZ836339 and KT156832). CD04 was found to carry the T440A mutation, which was also observed in two Western CPV-2c strains, one from Chile (MT585703) and one from the USA (FJ005236). In addition, four Xichang strains (XC02, XC05, XC06 and XC08) exhibited the Trp214Cys mutation and formed an independent small branch on the evolutionary tree. The CPV-2c strains from this study shared 98.8-100% nucleotide identity and 98.6-100% amino acid identity among themselves (Tables S3 and S4). Notably, 54.35% (25/46) exhibited 100% amino acid identity with CPV-2c reference strains reported in several Chinese regions, Nigeria, and Italy from 2014 to 2021.

### 3.3. NS1 Gene Phylogenetic Analysis of CBoV

**Figure 4:**
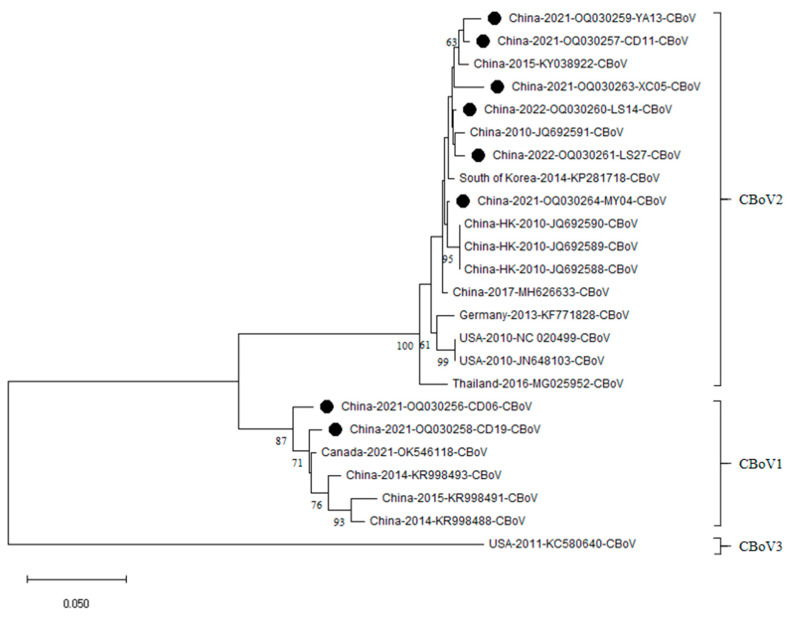
The evolutionary history was inferred using the Neighbor-Joining method. The optimal tree with the sum of branch length = 0.67599231 is shown. The percentage of replicate trees in which the associated taxa clustered together in the bootstrap test (1000 replicates) are shown next to the branches. The tree is drawn to scale, with branch lengths in the same units as those of the evolutionary distances used to infer the phylogenetic tree. The evolutionary distances were computed using the Kimura 2-parameter method and are in the units of the number of base substitutions per site. The analysis involved 24 nucleotide sequences. All positions containing gaps and missing data were eliminated. Black symbols indicate CBoV strains identified in the present study.

Phylogenetic analysis showed that the eight sequences from this study clustered into two distinct groups (Figure 4). Six of the strains were found within a major clade that contained 11 reference strains from Asia, America and Europe. The remaining two strains (OQ030256 and OQ030258) formed a well-supported subclade with four reference sequences from China (KR998493, KR998488 and KR998491) and Canada (OK546118). Based on their phylogenetic positions, all eight sequences were classified into two established CBoV subspecies: six strains belonged to CBoV2, and two strains (OQ030256 and OQ030258) were assigned to CBoV1. Notably, no strains from this study clustered with the CBoV3 subspecies.

### 3.4. VP2 Gene Phylogenetic Analysis of CbuV

**Figure 5:**
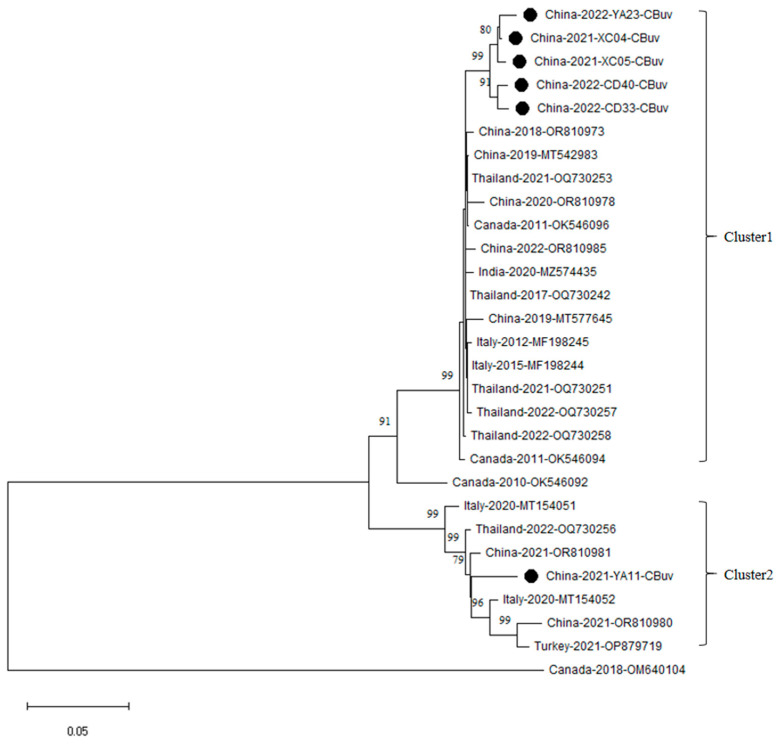
Phylogenetic analysis of CBuV based on VP2 gene sequences. The evolutionary history was inferred using the Neighbor-Joining method. The optimal tree with the sum of branch length = 0.73839789 is shown. The percentage of replicate trees in which the associated taxa clustered together in the bootstrap test (1000 replicates) are shown next to the branches. The tree is drawn to scale, with branch lengths in the same units as those of the evolutionary distances used to infer the phylogenetic tree. The evolutionary distances were computed using the Kimura 2-parameter method and are in the units of the number of base substitutions per site. The analysis involved 30 nucleotide sequences. All positions containing gaps and missing data were eliminated. Black symbols indicate CBuV strains identified in the present study.

Phylogenetic analysis showed that the six sequences from this study clustered into two distinct genetic groups (Cluster 1 and Cluster 2, Figure 5). Cluster 1 was a well-supported major clade that included five CBuV strains from this study (CD33, CD40, XC04, XC05 and YA23) and reference sequences from China, Thailand, Italy and India. Within this major clade, the five CBuV strains from this study formed a distinct, monophyletic subcluster with moderate support. In the cluster 2, the remaining one strain (YA11) formed a well-supported subclade with four reference sequences from Italy, China and Turkey.

## 4. Discussion

CPV is the primary cause of severe viral enteritis in dogs worldwide. In recent years, two novel parvoviruses (CBoV and CBuV) have been identified as recently discovered pathogens and are now widely recognized as common components of the canine enteric virome [5,6]. Notably, co-infections involving CPV, CBoV and CBuV have been reported, and these are considered to increase the complexity of viral enteritis [36,42]. Before this study, the epidemiology of CPV in Sichuan was limited by geographic coverage and sample size. Moreover, the presence and molecular characteristics of CBoV and CBuV in Sichuan had not been reported. This study examined 144 diarrheic dogs using PCR between 2020 and 2022 in Sichuan Province, exploring the characteristics of CPV, CBoV, and CBuV and analyzing their co-infection rates.

In the present study, we analyzed the detection rates and co-infection patterns of CPV-2, CBoV and CBuV in Sichuan. Our results showed CPV-2 was the most prevalent virus detected (33.3%) in diarrheic dogs across the five regions surveyed, indicating its status as a dominant and widespread enteric pathogen in Sichuan. The detection rates of CPV-2 in our study was lower than that reported in Shandong (62.9%), and Shanghai (40.78%), and East China (40.7%) [15,16,49]. Additionally, CBoV and CBuV were first detected in Sichuan, with positive rates of 5.56% (8/144) and 4.17% (6/144), respectively. Recent studies have indicated that CBoV has become endemic among dogs worldwide [5,7,31,33,35,37]. The detection rates of CBoV in our study was higher than that reported in Guangdong (0.24%) [31], but lower than in Heilongjiang (20%) [36]. It aligns closely with reports from Korea (9.6%) [33]. As to CBuV, only four surveys had analyzed the prevalence of CBuV in fecal samples from diarrheic in China before 2022. The detection rates of CBuV in our study (4.17%) was similar to that observed in Guangxi (6.25%) [39]. This is higher than in Henan (1.74%) and Anhui (4.4%) [43,44], but lower than that reported in Shanghai (42.51%) [40]. Furthermore, we identified four cases of co-infection (2.78%, 4/144), including one triple infection (CPV/CBoV/CBuV) in the Xichang City (XC05). This phenomenon is consistent with the previous studies. For example, Sun et al. found that 6.25% of canine samples tested positive for CBuV, all of which were co-infected with CPV [44]. Similarly, Yao et al. found that 37.5% (3/8) of CBoV-positive samples were co-infected with CPV [7]. In this study, most virus-positive dogs were unvaccinated or incompletely vaccinated, including 91.67% of CPV-2–positive dogs, 75.0% of CBoV-positive dogs, and all CBuV-positive dogs. Similarly, a recent epidemiological study in China reported that incomplete or insufficient vaccination coverage was closely associated with the high prevalence and sustained circulation of canine parvovirus [50]. Therefore, these observations further underscore the importance of timely and complete vaccination in preventing canine viral enteric infections.

The distribution of CPV genotypes shows significant geographical variation, which highlights their dynamic evolutionary characteristics. Our genetic analysis confirmed that the CPV-2c genotype is dominant in Sichuan, accounting for 95.8% (46/48) of CPV-positive strains. Previous studies in Sichuan Province have indicated that CPV-2a and CPV-new 2a were the predominant circulating strains between 2011 and 2020 [22,25,28,34]. However, our data from 2020 to 2022 clearly highlighted that CPV-2c was the most prevalent genotype. This finding is consistent with the national trend observed in the provinces of Henan and Jilin [19,21], further establishing CPV-2c as the dominant variant in China. Additionally, we further analyzed the amino acid variations in the VP2 protein of CPV-2 strains. All Sichuan strains in our study belonged to the ‘Asian CPV-2c’, characterized by VP2 substitutions (Phe267Tyr, Tyr324Ile, and Gln370Arg), which distinguish them from Western CPV-2c strains in terms of their phylogenetic classification [51]. Phylogenetic analysis demonstrated that the Asian clade is subdivided into Asia-1.1 and Asia-1.2, primarily due to the I447M substitution. Within the Asia-1.1 subclade, two strains (MY21 and CD20) carried the Ala5Gly mutation and showed close phylogenetic relationships with Sichuan isolates from 2016 and 2017, indicating the sustained circulation of CPV-2c with Ala5Gly in Sichuan. The Ala5Gly mutation was first reported in Beijing, China, in 2015 [52]. Statistical analysis of Chinese Ala5Gly mutant strains revealed that this mutation first appeared in isolates from 2013 and has increased in prevalence in recent years, with occasional detections in Italy and Nigeria [21]. Except for Ala5Gly, other key mutations also were observed in our study, such as Ile447Met and Thr440Ala. In the Asian 1.2, both our LS03 strain and the new Vietnamese CPV-2c strain carried the Ile447Met mutation. However, the Vietnam new CPV-2c strain also carried Ala5Gly, whereas LS03 did not. This may explain why they are in the same major branch, but on distinct sub-branches. A previous study found that Vietnamese CPV-2c strains had a unique non-synonymous Ile447Met mutation in VP2 genes, which was subsequently detected in Henan Province, China [21]. This suggests that the strain carried Ile447Met mutation may have been transmitted to China through the pet trade and population migration. Furthermore, we identified the Thr440Ala mutation in CPV-2c strains (CD04). At present, Thr440Ala mutation is only found in CPV-2a/2b and has been reported worldwide, including in Africa, India and China [53,54,55,56]. However, the detection of the Thr440Ala mutation in CPV-2c in our study suggests a possible genetic recombination event which deserves further investigation to understand its origin and implications.

Our study revealed the co-circulation of both CBoV-1 and CBoV-2 in Sichuan province. This finding contrasts with reports from Guangdong and Heilongjiang, where only CBoV-2 was detected [7,31], suggesting a geographical variation in the genetic diversity of CBoV across China. Regarding CBuV, our phylogenetic analysis identified two clusters, with the two Ya’an strains exhibiting significant genetic differences. Within cluster 2, our YA11 strain clustered with the Italian strain MT5402. Interestingly, the result is similar with the phylogenetic pattern observed in a study from Thailand, where two Thailand CBuV strains also formed a clade (subgroup B) with the same Italian MT5402 strain [57]. This specific genetic link between the Asian strains (from our study and from Thailand) and the Italian MT5402 strain suggests that they may have the same common ancestor.

This study has several limitations: (1) The modest sample size may limit the statistical power of our phylogenetic analysis. (2) The uneven sampling across regions (e.g., only 15 samples from Xichang) may not accurately reflect the prevalence and diversity of viruses across the entire study area. (3) Only diarrheic dogs’ samples (not including health dogs’ samples) were collected, which may introduce selection bias. Therefore, the detection rates reported here should not be interpreted as population-level prevalence but rather as the occurrence of viral infections among clinically affected dogs. Future studies including healthy control populations and population-based sampling would be necessary to assess statistical associations with diarrhea.

## 5. Conclusions

In this study, we have carried out a molecular characterization of CPV, CBoV and CBuV in dogs with enteric disease in Sichuan, China, with CPV-2c being the predominant pathogen (46 strains). Molecular and phylogenetic analyses confirmed that all CPV-2c strains belonged to the ‘Asian CPV-2c’ lineage, which has become the dominant genotype in Sichuan. Furthermore, the co-circulation of CBoV-1 and CBoV-2 reveals a greater genetic diversity of bocaviruses in Sichuan. This study is the first to report the identification of CBoV and CBuV in dogs in Sichuan province. Our findings highlight the increasing detection rates and genetic diversity of canine parvoviruses. Therefore, continuous monitoring the incidence and molecular characteristics of CPV-2c and these novel parvoviruses (CBoV and CBuV) is fundamental to controlling their spread.

## Figures and Tables

**Table 1:** Clinical information and infection status of virus positive samples.

Number	Source	Breed	AGE	Clinical Symptoms	Vacccination	Infected Virus			Genbank Number
						CPV	CBoV	CBuV	
CD03	Chengdu	Pomeranian	3 months	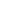 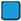 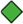 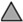	No	+	-	-	CPV:MZ857180
CD04	Chengdu	Chinese Field Dog	5 months	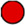 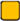 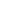 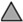	No	+	-	-	CPV:MZ857181
CD05	Chengdu	Chinese Field Dog	2 months	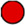 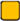 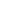 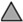	No	+	-	-	CPV:MZ857185
CD06	Chengdu	Poodle	7 years	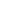 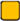 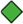	Complete	-	+	-	CBoV:OQ030256
CD08	Chengdu	Chinese Field Dog	4 months	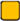 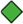 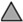	Incomplete	+	-	-	CPV:OP779632
CD09	Chengdu	Poodle	3 months	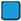 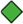 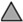	No	+	-	-	CPV:OP779633
CD10	Chengdu	Chinese Field Dog	2 months	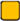 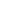 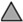	No	+	-	-	CPV:OP779634
CD11	Chengdu	Chinese Field Dog	4 months	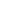 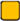 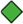	No	-	+	-	CBoV:OQ030257
CD14	Chengdu	Chinese Field Dog	16 months	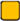 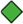 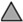	No	+	-	-	CPV:OP779635
CD19	Chengdu	Chinese Field Dog	1.5 months	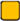 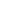 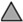	No	-	+	-	CBoV:OQ030258
CD21	Chengdu	Chinese Field Dog	2 months	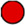 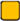 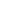 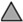	No	+	-	-	CPV:OP779636
CD30	Chengdu	Poodle	-	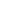 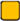 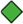	No	+	-	-	CPV:OP779637
CD33	Chengdu	Chinese Field Dog	2 months	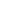 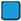 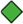 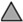 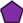	No	-	-	+	CBuV:OQ030264
CD38	Chengdu	Pomeranian	2 months	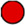 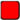 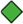 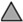	No	+	-	-	CPV:OP779638
CD40	Chengdu	Welsh Corgi	2 months	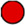 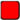 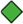 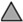 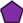	No	+	-	+	CPV:OP779639 CBuV:OQ030265
YA04	Ya’an	Golden Retriever	3 months	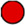 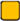 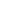 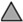	Incomplete	+	-	-	CPV:MZ857182
YA05	Ya’an	Welsh Corgi	2 months	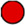 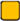 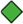 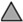	Incomplete	+	-	-	CPV:MZ857183
YA06	Ya’an	Labrador Retriever	3 months	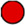 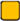 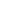 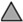	Incomplete	+	-	-	CPV:MZ857186
YA08	Ya’an	Chinese Field Dog	4 months	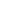 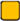 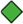	Incomplete	+	-	-	CPV:OP779640
YA10	Ya’an	Chinese Field Dog	3 months	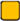 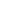 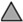	Incomplete	+	-	-	CPV:OP779641
YA11	Ya’an	Greyhound	2 months	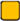 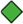 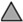	No	+	-	+	CPV:OP779642 CBuV:OQ030266
YA13	Ya’an	Chinese Field Dog	2 months	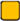 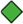 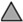	No	+	+	-	CPV:OP779643 CBoV:OQ030259
YA17	Ya’an	Chinese Field Dog	2 months	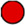 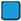 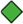 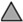	Incomplete	+	-	-	CPV:OP779644
YA18	Ya’an	Chinese Field Dog	9 months	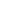 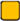 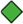 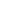 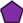	Incomplete	+	-	-	CPV:OP779645
YA23	Ya’an	Chinese Field Dog	3 monthes	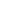 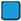 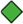 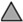 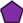	Incomplete	-	-	+	CBuV:OQ030267
YA25	Ya’an	Chinese Field Dog	2 months	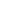 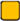 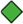 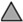 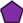	No	+	-	-	CPV:OP779646
YA27	Ya’an	Chinese Field Dog	2 months	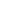 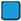 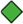 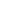 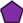	No	+	-	-	CPV:OP779647
YA29	Ya’an	Chinese Field Dog	2 months	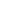 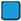 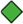 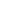 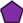	No	+	-	-	CPV:OP779648
YA32	Ya’an	Chinese Field Dog	2 months	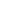 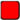 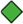 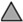 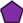	No	+	-	-	CPV:OP779649
LS03	Leshan	Golden Retriever	-	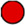 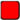 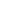 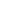 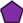	No	+	-	-	CPV:OP779650
LS07	Leshan	Chinese Field Dog	5 months	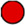 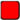 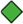 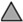 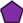	Incomplete	+	-	-	CPV:OP779651
LS08	Leshan	Chinese Field Dog	4 months	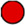 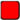 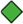 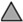 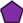	Incomplete	+	-	-	CPV:OP779652
LS09	Leshan	Chinese Field Dog	4 months	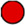 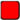 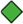 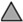 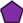	Incomplete	+	-	-	CPV:OP779653
LS14	Leshan	Welsh Corgi	2 years	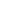 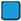 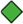 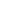 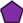	Complete	-	+	-	CBoV:OQ030260
LS20	Leshan	Poodle	3 months	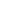 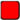 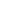 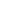 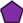	Incomplete	+	-	-	CPV:OP779654
LS21	Leshan	Golden Retriever	2 months	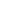 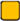 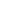 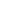 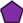	Incomplete	+	-	-	CPV:OP779655
LS27	Leshan	Poodle	2 months	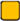 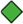 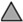	No	-	+	-	CBoV:OQ030261
LS29	Leshan	Chinese Field Dog	2 months	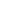 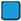 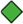 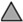 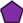	No	+	-	-	CPV:OP779656
MY01	Mianyang	Chinese Field Dog	3 months	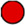 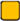 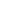 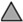	Incomplete	+	-	-	CPV:OP779657
MY02	Mianyang	Poodle	5 months	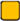 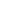 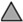	Incomplete	+	-	-	CPV:OP779658
MY04	Mianyang	Poodle	2 years	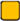 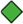 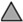	Incomplete	-	+	-	CBoV:OQ030262
MY05	Mianyang	Poodle	1 months	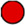 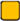 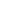 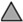	No	+	-	-	CPV:OP779659
MY06	Mianyang	Poodle	4 months	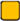 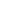 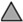	Incomplete	+	-	-	CPV:OP779660
MY07	Mianyang	Pomeranian	3 months	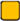 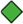 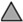	Incomplete	+	-	-	CPV:OP779661
MY09	Mianyang	Pug	3 months	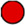 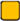 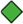 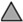	Incomplete	+	-	-	CPV:OP779662
MY14	Mianyang	German Pinscher	2 months	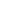 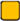 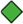	No	+	-	-	CPV:OP779663
MY15	Mianyang	Greyhound	10 months	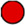 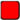 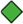 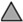 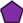	Complete	+	-	-	CPV:OP779664
MY17	Mianyang	Chinese Field Dog	2 months	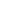 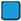 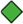 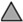 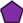	No	+	-	-	CPV:OP779665
MY18	Mianyang	Chinese Field Dog	3 months	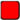 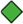 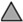	Incomplete	+	-	-	CPV:OP779666
MY19	Mianyang	Chinese Field Dog	2 months	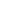 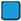 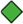 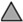 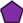	Incomplete	+	-	-	CPV:OP779667
MY20	Mianyang	Chinese Field Dog	3 months	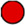 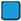 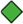 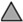 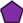	Incomplete	+	-	-	CPV:OP779668
MY21	Mianyang	Chinese Field Dog	6 months	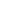 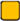 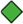 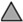 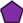	Complete	+	-	-	CPV:OP779669
XC02	Xichang	Golden Retriever	6 months	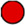 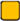 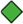 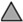	Complete	+	-	-	CPV:OP779670
XC04	Xichang	Poodle	4 months	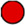 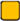 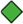 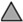	Incomplete	-	-	+	CBuV:OQ030268
XC05	Xichang	Poodle	3 months	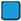 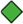 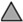	Incomplete	+	+	+	CPV:OP779671 CBoV:OQ030263 CBuV:OQ020369
XC06	Xichang	Welsh Corgi	3 months	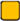 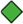 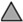	Incomplete	+	-	-	CPV:OP779672
XC08	Xichang	Pomeranian	24 months	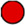 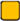 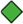 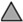	Complete	+	-	-	CPV:OP779673

Note. Fever 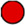; stool characteristics (bloody stool 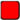, watery stool 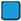, soft stool 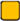); anorexia 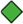; lethargy 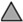; vomiting 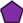.

## Data Availability

The original contributions presented in this study are included in the article and supplementary material. The gene sequences obtained in this study are openly available in the INSDC database under the following accession numbers: CPV (MZ857180-MZ857186, OP779632-OP779673, OQ030256-OQ030269), CBoV (OQ030256-OQ030263) and CBuV: (OQ030264-OQ030269, OQ020369). Further inquiries can be directed to the corresponding authors.

## Supplementary Materials

The following supporting information can be downloaded at: https://www.mdpi.com/article/10.3390/vetsci13010041/s1, Table S1: PCR protocols for the detection of CPV, CBoV, and CBuV in this study; Figure S1: original PCR images; Table S2: Amino acid variations in the VP2 protein of CPV-2 strains in this study and reference strain in this study; Table S3: Nucleotide identity among the CPV-2c strains in this study; Table S4: Amino acid identity among the CPV-2c strains in this study.
