# Supplementary figures and images for: Identification and Characterization of Canine Parvoviruses and Emergence of Canine Bocavirus and Bufavirus from Diarrheic Dogs in Sichuan Province, China
Source: Vet Sci. 2026 Jan 2;13(1):41. doi: 10.3390/vetsci13010041 (PMC12846450; doi:10.3390/vetsci13010041)

(A) PCR amplification of CPV.

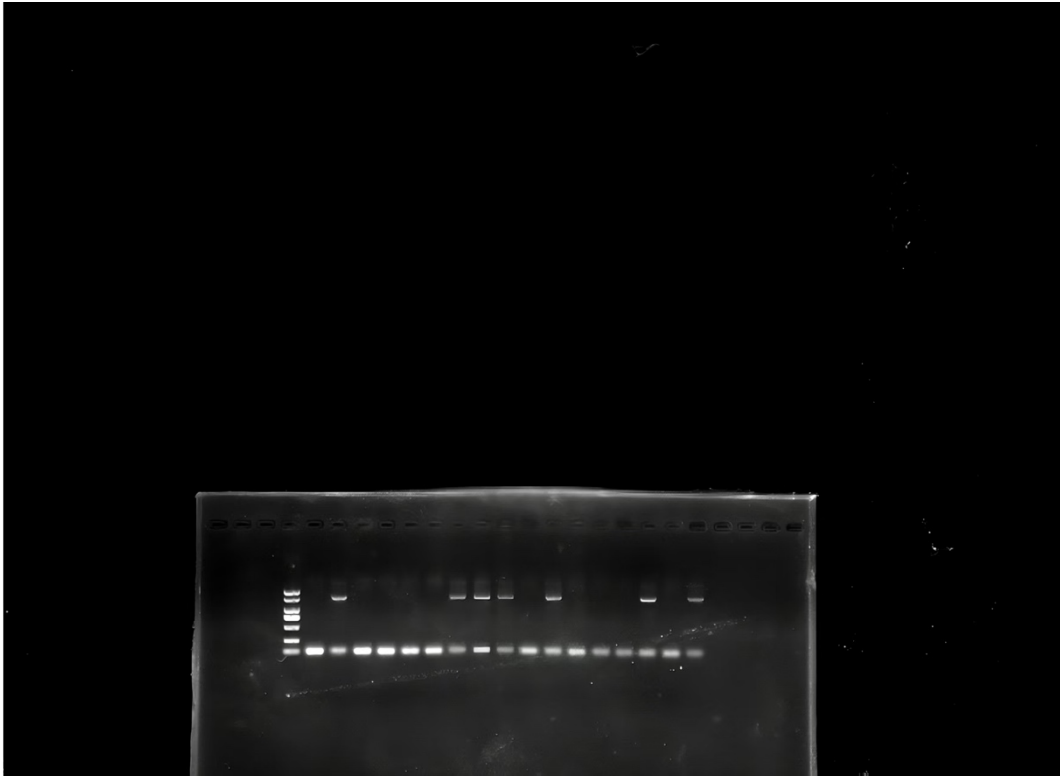

(B) PCR amplification of CBoV.

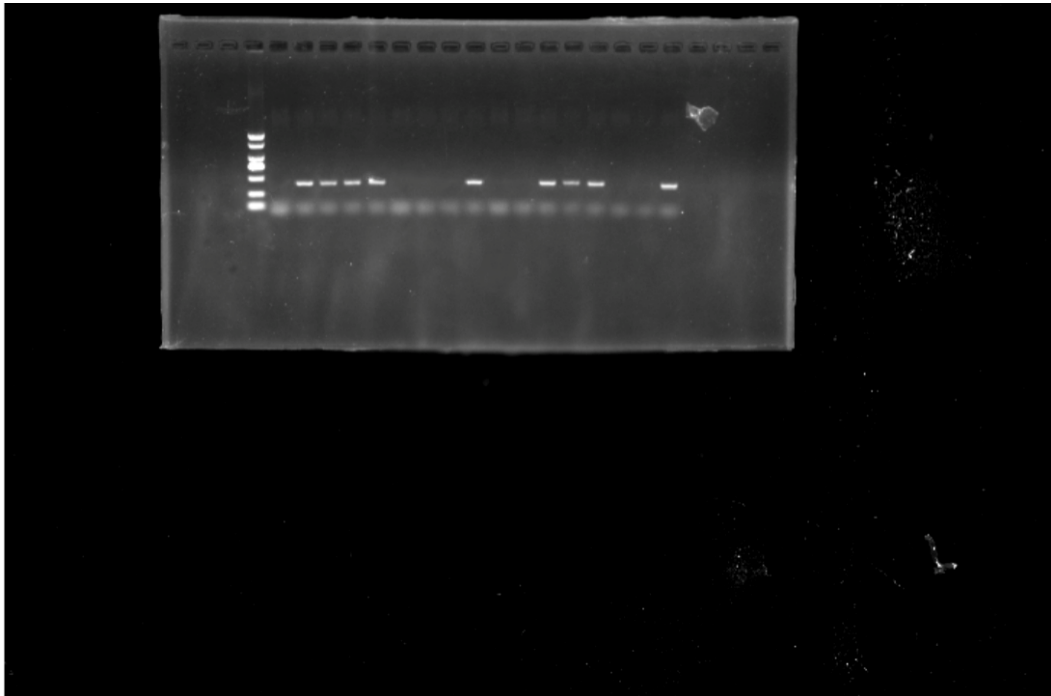

(C) PCR amplification of CBuV.

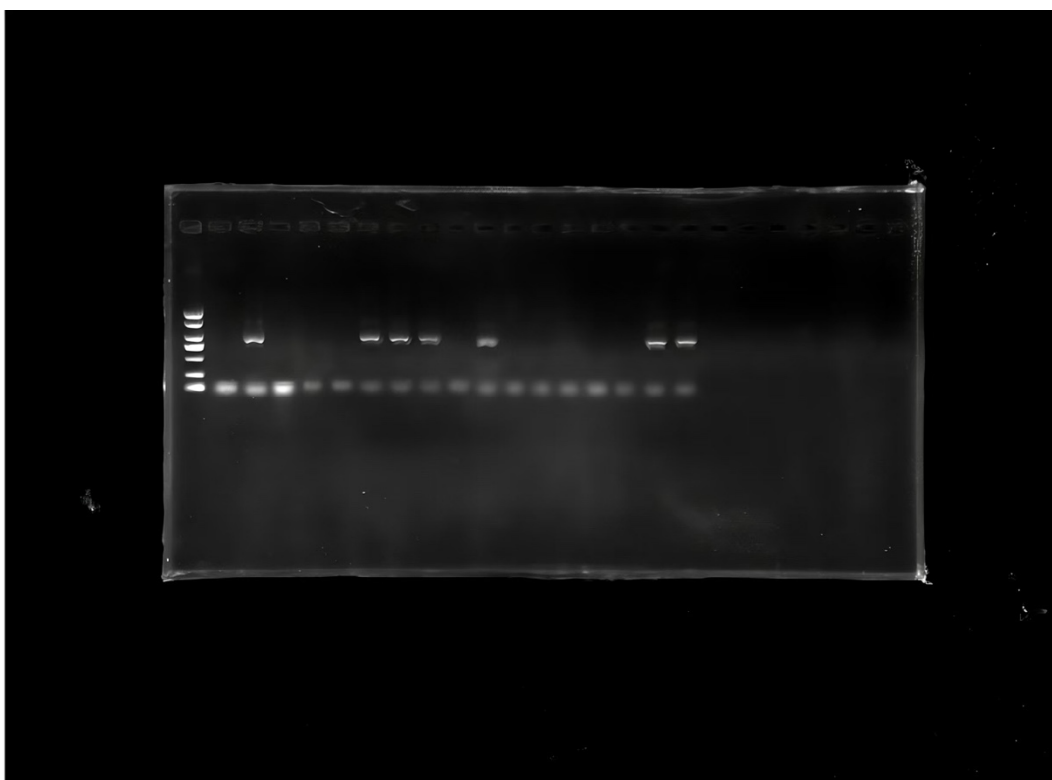

Supplement: Supplementary file 1 [file vetsci-13-00041-s001.zip › Figure S1. Original PCR image.pdf]
